# Tilt-induced changes in f-wave characteristics during atrial fibrillation: an experimental and computational investigation

**DOI:** 10.3389/fphys.2025.1498426

**Published:** 2025-06-13

**Authors:** Mostafa Abdollahpur, Chiara Celotto, Carlos Sánchez, Felix Plappert, Sten Östenson, Pyotr G. Platonov, Pablo Laguna, Esther Pueyo, Frida Sandberg

**Affiliations:** ^1^ Department of Biomedical Engineering, Lund University, Lund, Sweden; ^2^ BSICoS Group, Aragon Institute of Engineering Research (I3A) and IIS Aragón, University of Zaragoza, Zaragoza, Spain; ^3^ Centro de Investigación Biomédica en Red en Bioingeniería, Biomateriales y Nanomedicina (CIBER-BBN), Zaragoza, Spain; ^4^ Department of Internal Medicine and Department of Clinical Physiology, Central Hospital Kristianstad, Kristianstad, Sweden; ^5^ Department of Cardiology, Clinical Sciences and Center for Integrative Electrocardiology at Lund University (CIEL), Lund, Sweden

**Keywords:** atrial fibrillation, autonomic nervous system, ECG processing, f-wave frequency, parasympathetic regulation, respiratory modulation

## Abstract

**Introduction:**

This study explores transient and stationary effects of sympathetic and parasympathetic stimulation on f-wave characteristics in atrial fibrillation (AF) patients undergoing a tilt test. Transient phase is defined as the initial 2-minute interval following each postural change, reflecting immediate autonomic adaptation, whereas steady phase refers to the subsequent interval (from 3 minutes post-change until phase end) representing a stable autonomic state.

**Methods:**

Our primary aim is to investigate how the two branches of the autonomic nervous system (ANS) influence the f-wave frequency time series ( f(m) ). An analysis of f(m) in terms of the mean over time ( $F_{f}$ ) and the magnitude of respiration-modulated f(m) variations ( $\Delta F_{f}$ ) is conducted during baseline supine rest (B), head-down tilt (HDT) and head-up tilt (HUT). We analyzed data from a previous study in which 24 patients with persistent AF underwent a tilt test protocol, during which electrocardiograms (ECGs) were recorded. A model-based method was used to extract f(m) series from the ECG. Subsequently, an orthogonal subspace projection method was employed to quantify $\Delta F_{f}$ , considering an ECG-derived respiratory signal. Electrophysiological computational simulations were conducted on 2D and 3D human atrial persistent AF models to aid the interpretation of clinical findings. Various levels of cholinergic stimulation by acetylcholine and β -adrenergic stimulation by isoproterenol were tested in the models. The temporal modulation of acetylcholine, representing changes associated with respiration, was cyclically modeled using sinusoidal waveforms.

**Results:**

Analysis of the clinical data showed a decrease in $F_{f}$ from B to HDT and an increase from HDT to HUT. During HDT, $\Delta F_{f}$ initially increased in the transient phase before decreasing in the steady phase, then rose again during HUT. Analysis of the simulated data showed that increasing the concentration of Isoproterenol and/or acetylcholine resulted in a rise in $F_{f}$ . Additionally, the magnitude of $\Delta F_{f}$ was shown to be associated with the extent of acetylcholine fluctuation.

**Discussion:**

These results suggest that changes in f-wave frequency characteristics during HUT and HDT could be linked to changes in sympathetic activity, with parasympathetic activity possibly modulating the effects of sympathetic activity rather than being an independent driver of fibrillatory rate changes.

## 1 Introduction

Atrial fibrillation (AF) is the most prevalent cardiac arrhythmia characterized by irregular and rapid electrical impulses in the atria, leading to ineffective atrial contractions. This condition disrupts the heart’s normal rhythm posing serious health risks, including stroke, heart failure and decreased quality of life. As the incidence of AF continues to rise globally, understanding the mechanisms underlying this condition becomes increasingly important (Hindricks et al., 2021). However, despite advancements in the treatment of AF, identifying the most suitable therapy for each individual patient remains challenging (Hindricks et al., 2021; Joglar et al., 2024). The pathophysiology of AF is complex and involves various modulators that act via multiple mechanisms. Several studies have described a role of the autonomic nervous system (ANS) in the onset and perpetuation of AF (Gould et al., 2006; Chen et al., 2014; Vandenberk et al., 2023).

In healthy subjects, the sympathetic and parasympathetic branches of the ANS work together in coordination, with sympathetic activation increasing heart rate and contractility, and parasympathetic activation decreasing them. An imbalance in the activity of the ANS can lead to electrical instability in the heart, both acting as an AF trigger and contributing to the AF substrate required for the perpetuation of AF (Dimmer et al., 1998; Herweg et al., 1998; Fioranelli et al., 1999; Tomita et al., 2003; Chang et al., 2016). Therefore, assessing ANS activity in AF patients could be highly relevant, as inter-patient variability in ANS activity might contribute to explain the large differences in the effectiveness of anti-AF therapies between patients and might help in the development of personalized treatments.

Parasympathetic stimulation (PSS) triggers the release of the neurotransmitter acetylcholine (ACh). ACh binds to muscarinic receptors in atrial myocytes, ultimately causing the activation of a specific subset of potassium channels known as G protein-activated inwardly rectifying potassium channels. The ACh-activated potassium current, denoted as I_KACh_, is involved in the slowing of the impulse formation at the sinoatrial node (SAN), leading to bradycardia, and in the shortening of the action potential (AP) duration (APD) and the hyperpolarization of the resting membrane potential in atrial myocytes. These effects, which are concentration-dependent, enhance the dispersion of refractoriness in atrial tissue and render the atria more susceptible to reentry and AF by reducing the wavelength of reentry (Rohr et al., 1998). Conversely, sympathetic stimulation (SS) increases the firing rate of the SAN and causes a variety of changes in the activity of atrial myocytes by activating the β -adrenergic signaling cascade, which triggers the phosphorylation of various cellular substrates by protein kinase A (Xie et al., 2013). Sympathetic hyperactivity induces arrhythmias by different mechanisms, including the enhancement of Ca^2+^ influx into the cell and the promotion of spontaneous releases of Ca^2+^ from the sarcoplasmic reticulum (Chelu et al., 2009). The increase in the intracellular Ca^2+^ concentration can lead to an abnormal automaticity of atrial cells, manifested as early or delayed afterdepolarizations.

During AF, the P waves of the ECG, representative of atrial activation, are replaced with irregular and erratic waveforms of smaller amplitude denoted f-waves (Sornmo, 2018). With the aim to characterize the atrial electrical activity during AF, the f-wave frequency has received considerable clinical attention (Lankveld et al., 2014; Platonov et al., 2014). Particularly, the atrial dominant frequency, often referred to as the atrial fibrillatory rate and here denoted as $F_{\text{f}}$ , can be derived either through spectral (frequency domain) analysis by identifying the frequency presenting the highest peak in the power spectral density of the f-wave signal (Park et al., 2019), or through time-domain analysis of the ECG using model-based approaches (Henriksson et al., 2018a). Previous studies have shown that variations in ANS activity can result in f-wave frequency variations, an aspect which can not be studied by restricting the analysis to the dominant frequency $F_{\text{f}}$ , but requires detailed analysis of the f-waves frequency time series f(m) (Abdollahpur et al., 2021; Abdollahpur et al., 2022). Slow controlled breathing (0.125 Hz) in persistent AF (psAF) patients with permanent pacemaker has been shown to cause f(m) modulation at the respiration frequency (Stridh et al., 2003; Holmqvist et al., 2005), with the magnitude of the respiratory f-wave frequency modulation $\Delta F_{\text{f}}$ being reduced after vagal blockade (Abdollahpur et al., 2021). This suggests a link between f(m) modulation through respiration and parasympathetic activity.

Also, we conducted computational simulations to shed light on the mechanisms underlying those findings (Celotto et al., 2022; Celotto et al., 2024). We evaluated the role of the spatiotemporal release pattern of ACh, considered to vary in phase with inspiration and expiration, in modulating the f-wave frequency and reported that changes in the ACh concentrations were linearly correlated with changes in the f-wave frequency.

A common way used in clinical settings to assess autonomic function is the tilt test, which can provide valuable information about the ability of the ANS to regulate blood pressure and heart rate in response to postural changes (Freeman, 2006). A study examined changes in the f-wave frequency in response to changes in ANS activity induced by head-down tilt (HDT) and head-up tilt (HUT) in 40 patients with psAF and reported a reduction in the f-wave frequency during HDT compared to the baseline (B) and an increase in f-wave frequency in response to HUT (Östenson et al., 2017). Our further analysis of the data from that study showed that a change in the sign of the derivative of the population averaged f-wave frequency trends is noticeable after the initial 2 min of each 5-minute tilt phase (Plappert et al., 2022). This may indicate an initial transient response of the ANS (Fois et al., 2022), after which the system begins to return to baseline.

In this study, we investigated the relationship between autonomic influences and changes in $F_{\text{f}}$ , $\Delta F_{\text{f}}$ and heart rate $\left(F_{HR}\right)$ during HUT and HDT in psAF patients. To assess both the transient and the steady-state responses, we separately analyzed the ECG signals for the first 2 and the last 3 min of baseline, HDT and HUT.

To provide further insight into the autonomic modulation of changes in heart rate and f-wave characteristics, we developed 2D and 3D atrial computational models under different combinations of cholinergic and β-adrenergic stimulation. Preliminary results of this study were presented at the 2023 Computing in Cardiology conference (Celotto et al., 2023).

## 2 Materials and methods

The description of the clinical tilt test data is provided in Section 2.1. A brief description of the methods for ECG-based estimation of heart rate $F_{HR}$ , f-wave frequency $F_{\text{f}}$ , and the respiratory modulation quantified in terms of $\Delta F_{\text{f}}$ is provided in Sections 2.2–2.4 (Abdollahpur et al., 2022). The human atrial models with incorporation of autonomic effects and the numerical simulations are expanded from Section 2.5 to Section 2.8 (Celotto et al., 2023; 2024). Section 2.9 describes the statistical analysis performed to identify significant differences in the extracted characteristics between B, HDT and HUT.

### 2.1 Materials

The present study is based on analysis of data acquired in a previous study (Östenson et al., 2017) where patients admitted with persistent AF and planned for elective cardioversion were screened for participation. Patients with abnormal levels of thyroid hormones, severe renal failure requiring dialysis, or heart valve disease were excluded as well as were patients ablated for AF or on any of the Class I or Class III antiarrhythmic drugs. The 40 patients that were included in that study were all on anticoagulant therapy. Table 1 lists the clinical characteristics of this population. Standard 12-lead ECGs, sampled at 1 kHz, were recorded in three different phases: 5 minutes in the baseline supine rest (B) position (0°), 5 minutes in the HDT position (−30°), and 5 minutes in the HUT position (+60°), respectively. Details about the tilt test protocol can be found in Östenson et al. (2017). In this study, we analyzed ECG recordings from a subgroup of 29 patients, since 11 patients were excluded due to missing ECG data. This subgroup is consistent with the subgroup previously examined in Plappert et al. (2022).

**TABLE 1:** Clinical characteristics of the original study population (Östenson et al., 2017).

Variable	Value
Age (years)	64 ± 12
Gender (male/female)	25/15
AF duration (days)	90 (1–350)
Congestive heart failure	8
Hypertension	32
Ischemic heart disease	4
Diabetes mellitus	3
Beta-blockers	32
Digoxin	7

### 2.2 ECG preprocessing

The ECG preprocessing, ectopic beat detection, and QRST cancellation were performed using the CardioLund® ECG parser, developed by CardioLund Research AB, Lund, Sweden. The original ECG sampling rate of 1 kHz provides high-resolution suitable for QRS cancellation and f-wave extraction. However, this high sampling rate would lead to a considerable computational burden in subsequent f-wave analysis which is not needed given that f-wave frequency contents can be assumed constrained well below 25 Hz. Consequently, the extracted f-wave signals from lead V1 were resampled to 50 Hz to obtain an f-wave signal (x(n)) . Each recording was split into six segments; first 2 minutes of baseline rest (B1), last 3 minutes of baseline rest (B2), first 2 minutes of HDT corresponding to the transient phase (HDT1), last 3 minutes of HDT corresponding to the steady-state phase (HDT2), first 2 minutes of HUT corresponding to the transient phase (HUT1) and last 3 minutes of HUT corresponding to the steady-state phase (HUT2). The baseline segment was subdivided into B1 and B2 to improve the reliability of phase comparisons. Ectopic beats were disregarded for computation of the average heart rate $F_{HR}$ in each phase (B1, B2, HDT1, HDT2, HUT1, HUT2), by assessing the consistency in the shape and timing of each beat relative to the established normal template, identifying those that deviate significantly from the template as ectopic beats.

### 2.3 Estimation of the f-wave frequency trend from patients’ ECGs

A harmonic f-wave model was used to estimate the high-resolution trend of the f-wave frequency, f(m) (Henriksson et al., 2018b). This model represents the f-wave signal as the sum of a complex exponential signal with the fundamental frequency f and its second harmonic (Equation 1):

$$s\left(n;\theta \right)=\overset{2}{\underset{p=1}{\sum }}\;A_{p}e^{j\left(p2\pi fn/f_{s}+\phi_{p}\right)},$$ (1)

The model parameters $\theta ={\left[f\;A_{1}\;A_{2}\;\phi_{1}\;\phi_{2}\right]}^{T}$ were estimated by fitting the harmonic model to the analytic equivalent of x(n) , denoted as $x_{a}\left(n\right)$ , using a maximum likelihood approach. The fitting process was performed on 0.5-s segments of $x_{a}\left(n\right)$ , with 20 ms overlapping, to obtain the f-wave frequency trend f(m) sampled at 50 Hz. For the fitting, the fundamental frequency f was constrained to the interval $\left[f_{0}\pm 1.5\right]$ Hz, where $f_{0}$ corresponds to the maximum peak in the interval [4, 12] Hz of the Welch periodogram of each x(n) recording.

To assess the accuracy of the fitted model, a signal quality index S was computed (Equation 2):

$$S=1-\frac{\sigma_{\hat{e}}}{\sigma_{x_{a}}}$$ (2)

where $\sigma_{\hat{e}}$ and $\sigma_{x_{a}}$ denote the standard deviation of the model error and $x_{a}\left(n\right)$ , respectively. In this study, S was computed for non-overlapping 5-s segments. Only segments with S>0.3 were considered for further analysis, as previous studies have indicated that S larger than 0.3 is sufficient for accurate estimation of f(m) (Henriksson et al., 2018b). Details on the estimation of S can be found in Abdollahpur et al. (2022). If more than 10% of the segments in an ECG recording were considered insufficient for further analysis, the recording was completely excluded from further analysis. The f-wave frequency $F_{\text{f}}$ in each phase (B1, B2, HDT1, HDT2, HUT1, HUT2) was obtained by calculating the median of the corresponding f(m) trend within each phase.

### 2.4 Respiratory f-wave modulation

An orthogonal subspace projection technique (Varon et al., 2019) was used to extract respiration-related fluctuations in the f(m) series as proposed in Abdollahpur et al. (2022). This procedure was performed separately for each phase (B1, B2, HDT1, HDT2, HUT1, HUT2). For the extraction of an ECG-derived respiration signal, a lead-specific respiration signal was extracted from each ECG lead separately by employing the slope range approach (Kontaxis et al., 2019), which uses the difference between the maximum and minimum derivative in the QRS interval to quantify variations in the QRS morphology related to respiratory activity. Subsequently, a joint-lead respiration signal, r(m) , was obtained by employing the periodic component analysis (πCA) approach proposed by (Plappert et al., 2024); an estimate of the respiration rate $\left(F_{RR}\right)$ was also provided by this method.

With r(m) in a window of M samples, an (M−q)×q subspace projection matrix, V , was constructed, where each column consisted of a delayed version of r(m) (Equations 3, 4):

$$V=\left[r_{0},r_{1},\ldots ,r_{d},\ldots ,r_{q}\right],$$ (3)

$$r_{d}={\left[r\left(1+d\right),r\left(2+d\right),\ldots ,r\left(M-q+d\right)\right]}^{T}.$$ (4)

Then, f(m) was detrended by subtracting its mean, and the resulting signal, denoted as $\tilde{f}\left(m\right)$ , was projected onto the respiratory subspace generated by V to estimate the respiratory-related variations (Varon et al., 2019) (Equation 5):

$$f_{r}=V{\left(V^{⊺}V\right)}^{-1}V^{⊺}\tilde{f},$$ (5)

where $\tilde{f}={\left[\tilde{f}\left(1\right),\ldots ,\tilde{f}\left(M-q\right)\right]}^{T}$ . The vector $f_{r}={\left[f_{r}\left(1\right),\ldots ,f_{r}\left(M-q\right)\right]}^{T}$ represents the variations in the f-wave frequency series linearly related to the respiration signal r(m) .

The average peak amplitude in $f_{r}$ , considered as an estimate of the magnitude of the respiratory-induced f-wave frequency variations $\left(\Delta F_{\text{f}}\right)$ ^1^, was determined by (Equation 6):

$$\Delta F_{\text{f}}=\sqrt{\frac{2\cdot f_{r}^{⊺}f_{r}}{M-q}}.$$ (6)

Furthermore, to quantify the relative contribution of respiration to the variations in $f_{r}$ compared to $\tilde{f}$ , the relative power of $f_{r}$ , denoted as $P_{r}\left(\%\right)$ , was computed as (Equation 7):

$$P_{r}\left(\%\right)=\frac{f_{r}^{⊺}f_{r}}{{\tilde{f}}^{⊺}\tilde{f}}\times 100.$$ (7)

### 2.5 2D and 3D human atrial electrophysiological models

Computational models of human atrial electrical activity were built to run simulations that could aid in the interpretation of the clinical data recorded during tilt tests in patients with psAF. Stationary conditions with different levels of SS and PSS were simulated to gain insight into their contribution to f-wave frequency characteristics.

Human atrial electrical activity was simulated both in 2D square sheets of tissue as well as in 3D biatrial anatomical models representative of psAF. The 2D models represented square pieces of 7 × 7 ${\text{cm}}^{2}$ tissue, discretized in square elements of 200- μ m side. A uniform bottom-to-top fiber direction was assigned to the tissues. For the 3D biatrial models, the anatomy was in all cases defined as in Ferrer et al. (2015). The 3D anatomical model was discretized in a multi-layer mesh using linear hexahedral elements with an average edge length of 300 μ m. This resulted in a total of 754,893 nodes and 515,010 elements. The model included detailed regional descriptions of fiber direction and functional heterogeneity, considering eight regions with different electrophysiological properties (Celotto et al., 2024).

In the 3D models, we used longitudinal conductivity values and transverse to longitudinal conductivity ratios adapted from (Ferrer et al., 2015), as detailed in (Celotto et al., 2024). With these conductivity values in healthy atrial tissue (without fibrosis and without electrical remodeling), the total activation time (TAT) was 130 ms, which is consistent with values reported in the literature. Introducing electrical remodeling caused a slight increase in TAT to 134 ms. Additional incorporation of fibrosis elevated TAT to 180 ms, consistent with findings from (Wesselink et al., 2022) in patients with psAF. In the 2D models, we applied the same longitudinal conductivity values and transverse-to-longitudinal conductivity ratios as those used in the left atrial (LA) region of the 3D model. This configuration resulted in a longitudinal conduction velocity (CV) of 94.12 cm/s without fibrosis and 58 cm/s with fibrosis. These findings are consistent with values reported in previous studies involving patients with and without AF (Bayer et al., 2019).

The electrophysiological activity of human atrial cardiomyocytes was described by the Courtemanche AP model (Courtemanche et al., 1998). All the myocardial nodes in the 2D tissue mesh were assigned with the same electrophysiological characteristics representative of LA tissue. In the 3D models, the Courtemanche model was adapted to represent different atrial regions by varying the ionic current conductances as in Ferrer et al. (2015). These adjustments were made based on experimental observations regarding AP morphology and duration reported in several studies (Wang et al., 1990; 1993; Li et al., 2001; Cha et al., 2005; Seemann et al., 2006).

Parasympathetic stimulation effects were described by introducing the ACh-activated potassium current $I_{KACh}$ in the cellular models. The $I_{KACh}$ formulation was based on the study by Kneller et al. (2002) and subsequently updated as proposed by Bayer et al. (2019). The effects of β -adrenergic stimulation were modeled as proposed in González de la Fuente et al. (2013). In brief, the effects of the nonspecific β -adrenergic agonist Isoproterenol (Iso) were modeled by increasing the maximal conductances of the L-type calcium current (I_CaL_) and the slow delayed rectifier potassium current $\left(I_{Ks}\right)$ and by decreasing the maximal conductance of the transient outward potassium current $\left(I_{to}\right)$ , following the experimentally reported concentration-dependent conductance modulation curves reported in González de la Fuente et al. (2013). Specifically, for 0.005 μ M Iso, the conductance values were increased by 169% for g_CaL_, by 76.6% for g_Ks_, and decreased by 54.8% for g_to_. At the higher concentration of 1 μ M, these values were increased by 300% for g_CaL_, by 79.2% for g_Ks_, and decreased by 61.4% for g_to_.

Since the simulations aimed to replicate conditions similar to those observed in psAF patients, both electrical and structural remodeling (Sanders et al., 2003) was incorporated into the models, as follows. Electrical remodeling associated with psAF was represented by reducing the conductances of I_to_, I_CaL_ and the ultrarapid delayed rectifier potassium current (I_Kur_) by 50%, 70% and 50%, respectively, as in Courtemanche (1999), by increasing the conductance of the inward rectifier potassium current (I_K1_) by 100% (Dobrev et al., 2001), and by increasing the conductance of I_Ks_ by 100% (González de la Fuente et al., 2013). To incorporate psAF-induced structural remodeling in the 2D and 3D models, we introduced 20% diffuse fibrosis based on the ranges reported experimentally (Platonov et al., 2011). Specifically, we randomly selected 20% of the nodes based on a uniform distribution and we assigned them the MacCannell active fibroblast computational model (MacCannell et al., 2007). The fibroblast-fibroblast gap-junctional conductance was reduced 4-fold with respect to the myocyte-myocyte conductance. When myocytes were coupled to fibroblasts, the junctional conductance was linearly adjusted depending on the number of fibroblasts coupled to a myocyte.

### 2.6 Simulated ACh and Iso release patterns

In both the 2D and 3D models, 30% of nodes were randomly chosen to be either ACh- or Iso-release nodes. This resulted in four possible scenarios: nodes that released only ACh, nodes that released only Iso, nodes that released both Iso and ACh and nodes that released neither Iso nor ACh.

To model the respiratory modulation of ACh concentration, the temporal pattern of ACh release was modeled as cyclically varying following a sinusoidal waveform with a frequency equal to the average respiration frequency measured in patients (0.14 Hz), see Table 2. A mean ACh level of 0.05 μ M was considered, while two different peak-to-peak variation ranges of ACh ( Δ ACh) equal to 0.1 and 0.025 μ M, were tested, all of them laying within the ACh ranges used in previous studies (0.0 − 0.1 μ M) (Bayer et al., 2019). The effects of β -adrenergic stimulation were simulated by administration of Iso at spatially and temporally fixed concentrations of 0.0, 0.005 and 1.0 μ M.

**TABLE 2:** Results of the evaluated parameters $F_{\text{f}}$ , $F_{HR}$ , $F_{RR}$ , $\Delta F_{\text{f}}$ and $P_{r}$ in the tilt test phases B1, B2, HDT1, HDT2, HUT1, and HUT2.

Median (IQR)	B		HDT		HUT	
mean ± SD	B1	B2	HDT1	HDT2	HUT1	HUT2
$F_{\text{f}}$ (Hz)	6.74(6.36−7.30)	6.69(6.43−7.35)	$6.49\;{\left(6.32-7.10\right)}^{*,**}$	$6.55\;{\left(6.13-6.91\right)}^{**}$	$6.77\;{\left(6.42-7.27\right)}^{†,‡}$	$6.72\;{\left(6.48-7.22\right)}^{‡}$
$F_{HR}$ (bpm)	93.5(80−105)	89.75(80−104)	$90.25\;{\left(80-106\right)}^{**}$	$93.5\;{\left(82-107\right)}^{**,†}$	$98.5\;{\left(84.5-114\right)}^{†,‡}$	$96\;{\left(82-111\right)}^{\#}$
$F_{RR}$ (Hz)	0.13(0.11−0.16)	0.13(0.11−0.24)	0.15(0.12−0.21)	0.16(0.12−0.25)	0.12(0.11−0.14)	0.15(0.12−0.16)
$\Delta F_{\text{f}}$ (Hz)	0.077±0.028	0.070±0.025	$0.090\pm 0.038^{**}$	$0.063\pm 0.019^{†}$	$0.081\pm 0.022^{‡}$	$0.079\pm 0.024^{‡}$
$P_{r}$ (%)	2.84±1.69	$1.97\pm 1.04^{*}$	$3.22\pm 2.21^{**}$	$1.66\pm 0.85^{†}$	$2.86\pm 1.40^{‡}$	2.36±1.37

Significant differences are denoted by “*” for B1, “**” for B2, “†” for HDT1, “‡” for HDT2, and “#” for HUT1.

### 2.7 Numerical methods and simulations

To establish steady-state conditions, single cells were paced at a fixed cycle length (CL) of 800 ms over a period of 16 min (Celotto et al., 2024). The resulting steady-state values of the cellular model’s state variables were used to initialize the multi-cellular models.

In the 2D models, four stimuli at a CL of 800 ms were administered at the lower edge of the 2D tissue to pre-excite the model. Subsequently, a cross-stimulation protocol (S1-S2) was employed to induce a rotor. The first stimulus (S1) was applied at the lower edge of the tissue, while the second stimulus (S2) was applied onto a 3.5 by 3.5 cm square at the bottom right corner.

In the 3D whole-atria models, an S1-S2 protocol was applied to trigger arrhythmias too. The S1 stimulus was administered at a line connecting the region between the superior and inferior left pulmonary veins with the area between the right pulmonary veins. Subsequently, the S2 stimulus was applied parallel to the first one starting from the inferior left PV and covering only half of the length of the S1 line (Celotto et al., 2024).

Following the delivery of the S1 stimulus, the simulations were conducted for a duration of 24 s, and results are presented for the last 10 s.

In both the 2D and 3D simulations, the S1-S2 intervals varied mainly based on the underlying Iso concentration, ranging from 130 ms at Iso = 0 μ M, to 110 ms at the highest concentration of Iso = 1 μ M.

Electrical propagation in the atria was described by the monodomain model and solved with the Finite Element Method in combination with the operator splitting numerical scheme using the software ELVIRA (Heidenreich et al., 2010).

### 2.8 Estimation of the simulated atrial activation frequency trend

From the simulations, transmembrane voltage time series were extracted from 169 uniformly distributed points in the 2D tissue models and 223 points manually selected to be approximately uniformly distributed in the 3D whole-atria models. For each extracted point c , the time instant $t_{c,i}$ corresponding to the maximum upstroke velocity of the i -th action potential was determined.

The simulated instantaneous frequency, $f_{c}^{s}\left(m\right)$ , was computed by resampling the series $1/\left(t_{c,i+1}-t_{c,i}\right)$ to a sampling frequency of 10 Hz, for all beat indices i in the recording. The time series $f_{c}^{s}\left(m\right)$ was subjected to power spectral analysis. Spectral “peak-conditioned” selection was performed as in Bailon et al. (2006) so that the series whose spectrum was not sufficiently peaked were discarded. The frequency trend of this simulated signals, $f^{s}\left(m\right)$ , was computed as the mean over the remaining $f_{c}^{s}\left(m\right)$ series for all points c not discarded for the analysis [more details can be found in Celotto et al. (2024)].

$F_{\text{f}}^{s}$ was calculated as the median over time of $f^{s}\left(m\right)$ . $\Delta F_{\text{f}}^{s}$ was computed using the method described in Section 2.4, considering as the respiration signal a sinusoidal waveform of frequency equal to 0.14 Hz.

### 2.9 Statistical analysis

The Lilliefors test was employed to assess the normality of the data. Results for Gaussian-distributed variables are presented as mean ± std, while results for non-Gaussian-distributed variables are presented as median (lower quartile − upper quartile). The Wilcoxon signed-rank test was used to assess statistically significant differences in $F_{HR}$ , $F_{\text{f}}$ , $\Delta F_{\text{f}}$ , and $P_{r}$ between phases. To account for multiple comparisons, a Bonferroni correction was applied, adjusting the statistical significance threshold. The corrected significance level was set at p<0.05/n , where n is the number of pairwise comparisons made.

**FIGURE 1:**
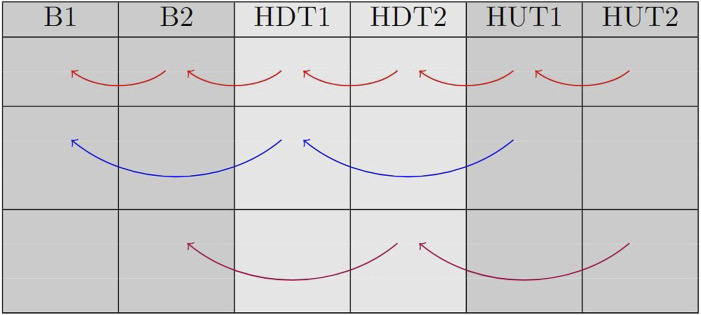
This table displays the comparisons that were made between phases. Red arrows represent comparisons between each sub-phase and the previous one; blue arrows represent comparison between transient phases; magenta arrows represent comparison between steady-state phases.

Figure 1 illustrates the comparisons that were performed in the study. Specifically, we compared each sub-phase (transient and steady-state) with the immediately preceding sub-phase. Additionally, we compared each steady-state phase with the previous steady-state one and each transient phase with the previous transient one.

## 3 Results

**FIGURE 2:**
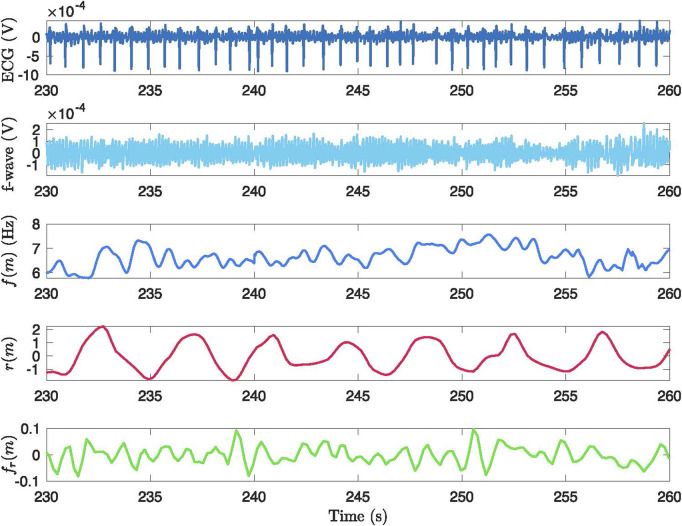
Example of the signal processing methodology applied to a 30-second segment from phase B2. The figure presents: (1) the original ECG signal, (2) the ECG signal after preprocessing and QRST cancellation, (3) the extracted f(m) signal over time, (4) the extracted respiratory signal r(m) , and (5) the projected frequency trend $f_{r}\left(m\right)$ after applying OSP.

An example of an original ECG signal and the corresponding extracted f-wave signal, estimated f-wave frequency trend f(m) , extracted respiratory signal r(m) , and respiratory-related f-wave frequency variations $f_{r}\left(m\right)$ , from a 30-s ECG segment during phase B2, are displayed in Figure 2.

**FIGURE 3:**
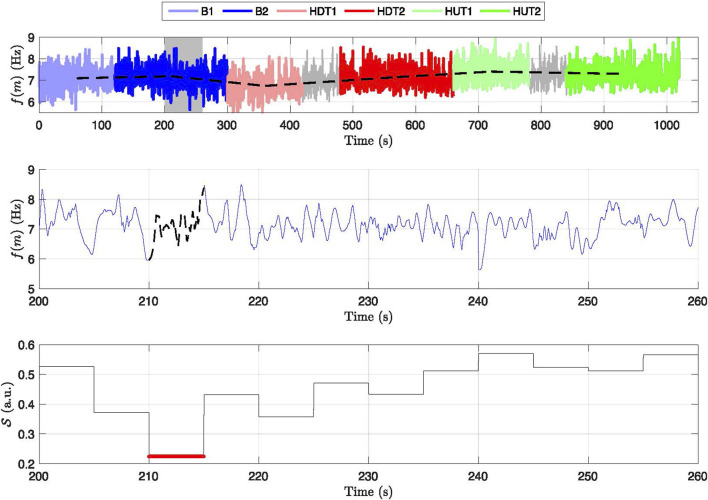
Frequency variation and signal quality across different phases for a subject. Top panel: Displays the frequency trend, f(m) , across baseline (B1, B2), head-down tilt (HDT1, HDT2), and head-up tilt (HUT1, HUT2) phases, with distinct colors marking the different phases. Dashed lines connect the mean f-wave frequency in each phase. Middle panel: Zoom in of a 60-s interval of f(m) corresponding to the shaded area in the top panel with dashed black line indicating segments where f(m) is excluded from further analysis. Bottom panel: Signal quality index S with red line indicating values below the threshold 0.3.

The estimated f-wave frequency trend f(m) from the entire recording of one patient is displayed in Figure 3, highlighting the variations in f(m) across phases (B1, B2, HDT1, HDT2, HUT1, HUT2). The signal quality index S , displayed for a 1-min subsegment of phase B1, shows that the f-wave signal quality is sufficient for analysis in a majority of the subsegment and pinpoints critical areas where the estimated f-wave frequency trend f(m) is considered unreliable.

### 3.1 Heart rate and f-wave frequency

Five out of the 29 patients were excluded entirely from further analysis based on the exclusion criteria outlined in Section 2.3 and thus the results are based on 24 patients. For the remaining 24 patients, 2.2% of the 5-s segments in B1, 1.7% in B2, 0.8% in HDT1, 2.9% in HDT2, 4.3% in HUT1, and 1.9% in HUT2 exhibited S<0.3 , leading to their exclusion from further analysis. Regarding the prevalence of ectopic beats in our analyzed segments, they accounted for approximately 1.07% of the total beats identified.

Table 2, first row, shows the results for $F_{\text{f}}$ in the first 2 min (transient) and last 3 min (steady state) of each tilt phase B, HDT and HUT. As can be seen from the table, there was a significant reduction in $F_{\text{f}}$ from B2 to HDT1 and no significant differences between HDT1 and HDT2. Conversely, a substantial rise from HDT2 to HUT1 was observed, again with no significant differences between HUT1 and HUT2. The second row in Table 2 shows results for $F_{HR}$ . A significant increase was observed from B2 to HDT1, from HDT1 to HDT2, from HDT2 to HUT1 and finally a significant decrease was observed from HUT1 to HUT2. Focusing on the steady state, a significant increase was observed from B2 to HDT2. Focusing on the transients, a significant increase was observed from HDT1 to HUT1.

**FIGURE 4:**
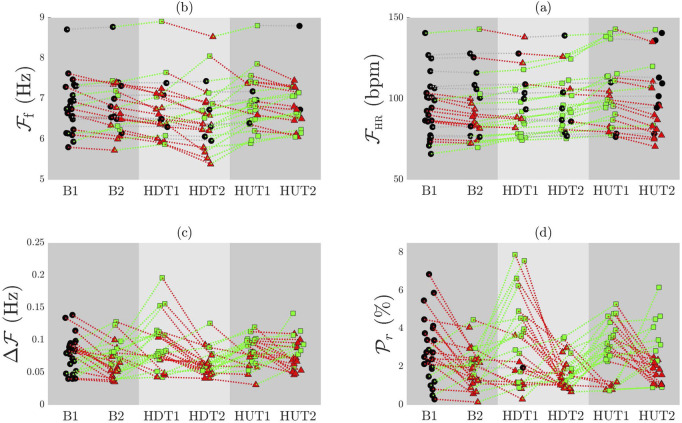
Individual patient trends for $F_{HR}$ **(a)**, $F_{\text{f}}$ **(b)**, $\Delta F_{\text{f}}$ **(c)**, and $P_{r}$ **(d)** across different phases of the tilt test. Responses relative to the preceding phase are illustrated by point shapes and line colors: increases are shown by green squares with green lines, decreases by red triangles with red lines, and minimal variations (less than 1%) by black circles with gray lines.

The subplots (a) and (b) of Figure 4 show the values of $F_{HR}$ and $F_{f}$ for each patient during each phase segment. The colors and shape of the points display the individual behavior of each patient in terms of increase (green squares), decrease (red triangles) and minimal variation (black circles, differences thives 1%) with respect to the previous phase segment, for $F_{\text{f}}$ and $F_{HR}$ , respectively. The behavior of $F_{HR}$ and $F_{\text{f}}$ notably varies among individuals. Note that heart rate $F_{HR}$ is expressed in bpm for consistency with other works and clinical conventions. However, it can be easily converted to Hz by dividing by 60: $F_{HR}$ (Hz) = $F_{HR}$ (bpm)/60. This conversion highlights the significantly higher frequency of f-waves compared to R-peaks repetition, i.e., the heart rate.

### 3.2 Respiration related f-wave variation and its relative power

The third row of Table 2 presents the results for the respiration rate $F_{RR}$ in each of the analyzed phase segments. There were no significant changes in respiration rate between the tilt phases. The fourth row of Table 2 shows the results for $\Delta F_{\text{f}}$ . There was a significant increase in $\Delta F_{\text{f}}$ from B2 to HDT1 and a significant decrease from HDT1 to HDT2. Also, there was a significant increase from HDT2 to HUT1. Comparing steady-states, there are no significant differences between phases, while considering transients there is a significant increase from HDT2 to HUT2. The last row of Table 2 shows the results for $P_{r}$ , indicating a significant decrease from B1 to B2, a significant increase from B2 to HDT1, a significant decrease from HDT1 to HDT2 and, finally, a significant increase from HDT2 to HUT1, similar to what was observed for $\Delta F_{\text{f}}$ .

The subplots (c) and (d) of Figure 4 illustrate the comparison of $\Delta F_{\text{f}}$ and $P_{r}$ in each patient for each phase segment. The colors and shape of the points display the individual behavior of each patient in terms of increase (green squares), decrease (red triangles) and minimal variation differences thives 1%) with respect to the previous phase (black circles), for $\Delta F_{\text{f}}$ and $P_{r}$ , respectively. Both $\Delta F_{\text{f}}$ and $P_{r}$ exhibited heterogeneous behavior across different patients.

### 3.3 The f-wave frequency analysis from computational simulations

**FIGURE 5:**
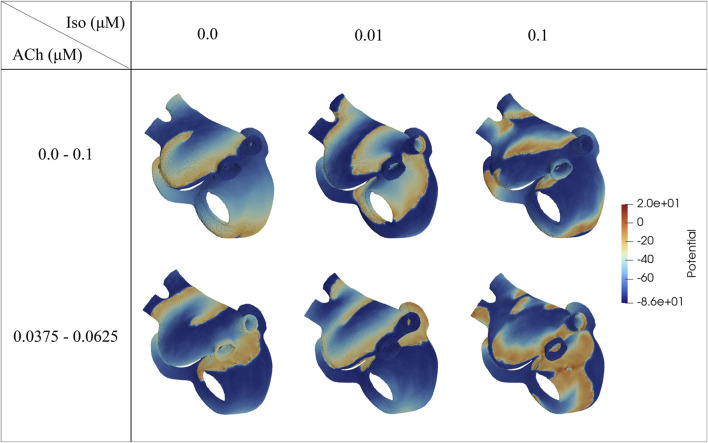
Voltage maps representative of the induced fibrillatory pattern after application of S1-S2 stimulation. The ACh values represent the range of variation ( Δ ACh). All snapshots were taken at the $20^{th}$ second from rotor initiation.

In the 2D tissue models, a single stable rotor was initiated after application of the S1-S2 protocol, while in the 3D biatrial models, S1-S2 stimulation was able to generate multiple stable rotors, as illustrated in Figure 5 and in the videos provided in the Supplementary Material. An increase in the number of stable rotors (from 1 to 3) was observed when adding 0.005 or 1 μ M Iso to ACh varying from 0 to 0.1 μ M. When Δ ACh was 0.025 μ M, the addition of 1 μ M Iso increased the number of rotors from 1 to 5.

**FIGURE 6:**
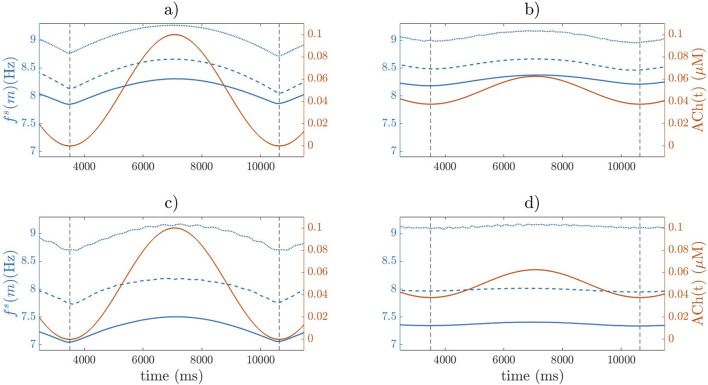
$f^{s}\left(m\right)$ (blue) and ACh (t) (red) from 2D simulations [panels **(a)** and **(b)**] and 3D biatrial simulations [panels **(c)** and **(d)**]. Solid, dashed, and dotted lines represent 0.0, 0.005, and 1 μ M Iso, respectively.

Table 3 reports the values of $F_{\text{f}}^{s}$ and $\Delta F_{\text{f}}^{s}$ for the different simulations. Figure 6 shows the temporal evolution of $f^{s}\left(m\right)$ compared to ACh concentration for each case. Additionally, Supplementary Figure S2S in the Supplementary Material displays the APD at 90% repolarization (APD_90_) over time across different locations within the tissue for the 3D simulations. 2D and 3D biatrial simulations render qualitatively and quantitatively comparable results in terms of $f^{s}\left(m\right)$ , with $F_{\text{f}}^{s}$ ranging from 7.12 Hz to 9.12 Hz in the different simulated cases. It can be observed that an increase in the minimum ACh level (from 0 μ M to 0.0375 μ M, corresponding to Δ ACh = 0.025 μ M) resulted in slightly higher $F_{\text{f}}^{s}$ values, with increases of 0.04 − 0.32 Hz in both the 2D and 3D simulations.

**TABLE 3:** $F_{\text{f}}^{s}$ and $\Delta F_{\text{f}}^{s}$ (Hz) computed from 2D and 3D simulations.

ACh release 0.14 Hz		2D			3D			Mean values	
		Iso ( μ M)			Iso ( μ M)				
ΔACh		0.0	0.005	1.0	0.0	0.005	1.0		
0.1	$F_{\text{f}}^{s}$	8.02	8.35	8.97	7.12	8.25	8.80	$\bar{F_{\text{f}}^{s}}$	8.25
	$\Delta F_{\text{f}}^{s}$	0.14	0.14	0.14	0.13	0.13	0.13	$\bar{\Delta F_{\text{f}}^{s}}$	0.13
0.025	$F_{\text{f}}^{s}$	8.20	8.51	9.01	7.37	8.50	9.12	$\bar{F_{\text{f}}^{s}}$	8.37
	$\Delta F_{\text{f}}^{s}$	0.03	0.03	0.03	0.03	0.03	0.03	$\bar{\Delta F_{\text{f}}^{s}}$	0.03

β -adrenergic stimulation via Iso significantly increased $F_{\text{f}}^{s}$ in all cases, with effects dependent on the concentration used (Table 3). In both the 2D and 3D models, an Iso concentration of 0.005 μ M resulted in an increase in $F_{\text{f}}^{s}$ ranging from 0.31 to 1.13 Hz compared to the control condition (no Iso). At a higher Iso concentration of 1 μ M, the increase was more pronounced, ranging from 0.81 to 1.75 Hz (Figure 6).

Regarding the variations in $\Delta F_{\text{f}}^{s}$ with ACh and Iso, we found that $\Delta F_{\text{f}}^{s}$ was mainly dependent on the range of ACh concentration variation, with its magnitude augmenting as the range increased, while Iso played a less relevant role. For the lowest ACh variation range of 0.025 μ M, $\Delta F_{\text{f}}^{s}$ was around 0.03 Hz. For the largest ACh variation range of 0.1 μ M, $\Delta F_{\text{f}}^{s}$ varied from 0.13 Hz (for the 2D cases) to 0.14 Hz (for the 3D cases).

## 4 Discussion

In this study, we analyzed ECGs of psAF patients undergoing a tilt table test and we computationally simulated the electrophysiology of the human atria to assess the relationship between ANS activity and f-wave frequency characterization.

We decided to analyze phases by distinguishing between transient and steady-state responses to postural changes in patients. In the study by Fois et al. (2022), the transient phases lasted approximately 1–1.5 min. In our dataset, these phases seemed to conclude around the 2-min mark. To avoid potentially overestimating the transient effects, we opted to consider slightly longer transient durations potentially including some of the steady-state.

Based on the analysis of the clinical data, substantial changes in $F_{\text{f}}$ were observed across B, HDT and HUT, both in transient and steady states. There was a decrease in $F_{\text{f}}$ transitioning from B to HDT, followed by an increase when transitioning from HDT to HUT. A similar behavior was observed in $F_{\text{HR}}$ . The most pronounced change in $\Delta F_{\text{f}}$ occurred during the HDT phase, with a significant increase from B2 to HDT1 and a significant decrease from HDT1 to HDT2. During the HUT phase, $\Delta F_{\text{f}}$ increased from HDT2 to HUT1 and then stabilized. A similar behavior was observed for $P_{r}$ .

Generally, the behavior of $F_{HR}$ and $F_{\text{f}}$ was shown to largely vary among individuals, with a relevant number of cases deviating from the overall trend. Inter-patient variability may arise from physiological differences among individuals, such as underlying health conditions, genetic predispositions and individual pharmacokinetic profiles, as well as from the use of β -blockers.

Through simulations, we found that increasing or decreasing the Iso concentration caused a corresponding increase or decrease in $F_{\text{f}}^{s}$ . Additionally, increasing the minimum ACh concentration led to a reduction in APD and resulted in a slight increase in $F_{\text{f}}^{s}$ (Supplementary Figure S1 in the Supplementary Material). Moreover, the level of ACh variation was found to be correlated with $\Delta F_{\text{f}}^{s}$ , as already shown in Celotto et al. (2024). Regarding the effects of the respiratory frequency, we only simulated a frequency of 0.14 Hz, corresponding to the average respiratory frequency of the patients in the various phases. Based on our previous study, we expect that variations in the respiratory rate within the 0.125 − 0.33 Hz range would not significantly affect $F_{\text{f}}^{s}$ , $\Delta F_{\text{f}}^{s}$ , or phase matching between ACh concentration variation and f(m) (Celotto et al., 2024).

A large number of studies have postulated an increase in SS during HUT in subjects in sinus rhythm (SR) (Cooke et al., 1999; Furlan et al., 2000; Whittle et al., 2022). This could be explained by the fact that during the transition to the HUT position, there is a sudden decrease in venous return, prompting a compensatory response from the sympathetic nervous system to maintain blood pressure. In our study, this observation is further reinforced in the context of AF. Specifically, the increase in $F_{\text{f}}$ that we observed in response to the HUT maneuver in AF patients is consistent with the increase in $F_{\text{f}}^{s}$ that we measured for increased Iso in our simulations.

Although there is scarce research on the autonomic effects during HDT, some studies in SR have associated the slowing of $F_{HR}$ during HDT with both an increase in PSS and a decrease in SS (Whittle et al., 2022; Porta et al., 2014). Only one study reported a decrease in both PSS and SS (Malhotra et al., 2021). In the case of AF or atrial flutter, some studies have linked the observed outcomes to an increase in PSS (Mase et al., 2008; Östenson et al., 2017). The findings of this work are not fully in line with those studies in AF, since PSS, causing a shortening of the effective refractory period, actually leads to an increase in $F_{\text{f}}^{s}$ , as reported in the literature and confirmed by our simulations (Sarmast, 2003). In our observations, there is a slight reduction in $F_{\text{f}}$ when going from B2 to HDT1 and no significant difference from HDT1 to HDT2. However, patients exhibit heterogeneous behavior, with 25% of patients showing an increase, 50% showing a decrease in $F_{\text{f}}$ and 25% showing no substantial change during HDT1 with respect to B2 and HDT2 with respect to HDT1, with larger inter-subject variability observed during HDT2. Simultaneously, in the majority of patients (75%), an increase in $\Delta F_{\text{f}}$ was observed during HDT1, which may indicate an increased parasympathetic predominance. Based on the results of our study, these HDT-induced changes could be explained by a reduction in SS and an increase in PSS predominance. In the case of $F_{\text{f}}$ , the reduction in SS and the increase in PSS may be balanced in some cases and not in others, leading to either an increase or a decrease in $F_{\text{f}}$ depending on which effect is predominant (if the extent of SS decrease predominates over the PSS increase, a reduction in $F_{\text{f}}$ would be expected, and vice versa).

During HDT2, we observed a decrease in $\Delta F_{\text{f}}$ compared to its value in HDT1, followed by an increase in $\Delta F_{\text{f}}$ during HUT1. This behavior could be attributed to the differing time course of PSS and SS effects. Parasympathetic activation affects the heart rate almost immediately and briefly, with inputs occurring every 2–4 s. In contrast, SS has longer latency effects, receiving inputs every 20–40 s and producing effects that last longer (Pizzo et al., 2022; Nair et al., 2023). Additionally, the continued stimulation by ACh might lead to the build-up of inhibitory substances that counteract its effects. This could dampen the $\Delta F_{\text{f}}$ response even while $F_{\text{f}}$ remains elevated. This is supported by the fact that the release of ACh in the mammalian heart has been reported to be modulated by a negative feedback mechanism (Wetzel and Brown, 1985; Manabe et al., 1991).

The inclusion of simulations in our study provided useful insights into the expected direction of the changes in the mean f-wave frequency and the magnitude of respiratory modulation during PSS and SS.

We did not incorporate studies involving direct microneurography measurements of sympathetic activity or in vivo ACh concentration changes during maneuvers such as tilt testing for model validation, as we could not identify studies specifically linking tilt testing, microneurography, and AF. Instead, we based our validation on more global electrophysiological markers, such as CV and total atrial activation time (Bayer et al., 2019; Sanders et al., 2003), as well as f-wave frequencies (Stridh et al., 2003; Holmqvist et al., 2005), which have been extensively studied in relation to autonomic modulation and AF.

The simulation methods employed in this study build upon well-established modeling approaches that have been previously validated in the literature. The Courtemanche model and its adaptations have been widely used to simulate atrial electrophysiology, including the effects of autonomic modulation (Courtemanche et al., 1998). The ionic current modifications used to implement the effects of ACh and β -adrenergic stimulation on atrial electrophysiology were based on experimentally characterized data (Kneller et al., 2002; González de la Fuente et al., 2013).

The observed discrepancies in the absolute values in clinical data and simulations (around 2 Hz in $F_{\text{f}}$ and 0.1 − 1 Hz in $\Delta F_{\text{f}}$ ) could be attributed to various factors, which can arise from limitations in either the clinical data or the simulations.

In this regard, we performed one additional simulation at the 2D level (Supplementary Figure S1 in the Supplementary Material). Particularly, we reduced the level of psAF electrical remodeling by 50%, resulting in an increase in APD_90_ of the baseline AP (no ACh, no Iso) of 25%. These modifications led to a reduction in $\Delta F_{\text{f}}$ of 0.85 Hz. However, with the longer APD_90_, it would have been more difficult to establish long-lasting rotors in the 2D tissue. Nevertheless, we believe that the qualitative conclusions remain valid, and that the observed differences can be partially attributed to generally longer APs in the patients. In this sense, matching the dominant frequency observed in simulations and clinical recordings may be useful to estimate the degree of electrical remodeling in each patient.

Furthermore, while in the simulations the modulation of the fibrillatory rate is determined only by ACh and Iso, in the clinical signals the changes in the f-wave frequency characteristics in response to HDT and HUT can possibly be attributed to additional factors beyond the ANS modulation. Among such factors, mechanical stretch and mechano-electrical feedback should be considered, as they have been reported to exert significant contribution to atrial electrical activity in patients with atrial flutter (Mase, et al., 2009; Ravelli et al., 2008; Waxman et al., 1991). A study conducted by Waxman et al. (1991) examined various interventions, including passive upright tilting, the strain phase of the Valsalva maneuver and expiration, and all of them were found to reduce the cardiac size. Interestingly, regardless of the autonomic activity, these interventions were found to independently increase the rate of atrial flutter. Similarly, Ravelli et al. (2008) found that acute atrial stretch caused by ventricular contractions and respiratory movements resulted in a shortening of the atrial flutter CL in humans. Importantly, even after blocking autonomic influences, oscillations in the atrial flutter CL were still present, further supporting the idea that factors beyond autonomic activity contribute to these oscillations.

The simulation results suggest that impaired sympathetic activity leads to a reduced increase in $F_{\text{f}}$ in response to HUT, while impaired parasympathetic activity results in lower $\Delta F_{\text{f}}$ values. The results from the analysis of clinical data reveal moderate changes in $F_{\text{f}}$ and low $\Delta F_{\text{f}}$ values, potentially indicating impairments in both sympathetic and parasympathetic activity, consistent with the clinical characteristics of the study cohort (Table 1).

From a clinical point of view, by elucidating the specific effects of sympathetic and parasympathetic activity on f-wave frequency modulation, clinicians could tailor pharmacological interventions targeting the ANS more effectively. For example, medications that selectively modulate sympathetic or parasympathetic activity could be prescribed based on an individual patient’s autonomic profile, potentially leading to improved rhythm control and symptom management (Vandenberk et al., 2023; Chen et al., 2014). Additionally, autonomic modulation of f(m) could serve as a marker for stratifying patients based on their risk of AF progression or complications. Identifying these patients early on could prompt more intensive monitoring and intervention strategies to mitigate their risk. Finally, insights into autonomic influences on f(m) modulation could also inform personalized lifestyle interventions aimed at reducing AF burden and improving overall cardiovascular health.

### 4.1 Limitations

Some limitations of this study should be acknowledged to provide direction for further work.

One of the main limitations of the present study is the small sample size. The results suggest that changes in $F_{\text{f}}$ and $\Delta F_{\text{f}}$ in response to HUT and HDT are moderate, with considerable interpatient variability. Consequently, these findings should be validated in a larger study population.

This study analyzed ECG recordings from a subset of 24 patients from an original cohort of 40 patients (Östenson et al., 2017). The exclusion was based on the availability and quality of ECG signals: 11 patients were excluded due to missing ECG recordings, and 5 additional patients were excluded due to insufficient ECG signal quality for f-wave analysis. Although excluding low-quality ECGs improves the reliability of the results, it also limits the representativeness of our sample in relation to the full 40-patient cohort (cf. Table1).

The dataset did not provide access to individual patient data, such as age, sex, AF duration, comorbidities and use of drugs. These factors are known to influence the ANS, and hence lack of detailed patient information hampers a deeper exploration of how these factors might interact with the autonomic responses measured, potentially affecting the interpretation and applicability of our results.

Given that 80% of the original 40-patient cohort were diagnosed with hypertension and treated with β -blockers, it is highly likely that the majority of the 24 patients analyzed share these characteristics. However, without detailed individual-level data, we cannot explicitly confirm these conditions for each patient included in our analysis. These characteristics of the study cohort may limit the generalizability of our findings across the broader population of AF patients, potentially biasing our results toward individuals with more pronounced autonomic disturbances associated with hypertension and the use of β -blockers.

Another limitation of this study is the absence of a direct ground truth measurement for respiration, as we did not use an independent reference method (e.g., spirometry or respiratory belts) to validate the ECG-derived respiratory signal. While the ECG-derived respiratory signal has been widely used in prior studies (Kontaxis et al., 2019) and provides valuable insights into respiration-related cardiac modulation, it remains an indirect estimate, and potential inaccuracies cannot be entirely ruled out. Additionally, the observed respiratory rate (0.13 Hz) is lower than typical resting respiration rates. Several physiological factors, including the use of beta-blockers, the supine position during tilt-table testing, and the controlled quiet room environment, likely contributed to a slower spontaneous breathing rate. Despite these plausible explanations, the lack of direct respiratory measurements prevents us from direct verification of this effect. Future studies incorporating simultaneous direct respiratory monitoring would help validate and refine the precision of the ECG-derived respiration analysis in similar patient populations.

Focusing on the simulations, due to a lack of reported knowledge on the spatial distribution of sympathetic and parasympathetic innervation in the atria, we simply considered a random distribution of an equal number of sympathetic and parasympathetic nodes, to provide some evidence for the effects of the cholinergic and β -adrenergic stimulation.

Our computational models are deterministic and based on averaged patient data. Thus, they do not reproduce all the spectra of inter-patient variability but are representative of a mean psAF patient. Particularly, the use of a single anatomical model and a single model describing cellular electrophysiology may not have fully captured the inter-patient variability observed in the clinical scenario. Further investigations using other computational AP models with different steady-state APD values, as well as using populations of models, could be conducted to assess the impact of AP properties on f-wave frequency characterizations. Furthermore, AF-related structural remodeling of the atria may present with various alterations such as an enlarged atrial chamber, hypertrophy of cardiomyocytes, increased mismatch between epicardial and endocardial myofibers’ orientations, changes in atrial wall thickness and, notably, an increased amount of fibrotic or connective tissue (Wyse et al., 2014; Schotten et al., 2011; Heijman et al., 2016). We represented psAF-related structural remodeling by a combination of gap junction remodeling, modeled through tissue conductance reduction in fibrotic regions, and fibroblast proliferation. Future studies incorporating different degrees of fibrosis as well as other psAF characteristics not accounted for in our model could delve deeper into the collective impact of these factors on $F_{\text{f}}$ and $\Delta F_{\text{f}}$ .

Finally, our current computational models do not allow us to assess the effects of ACh and Iso on $F_{HR}$ . However, future studies could incorporate a network model of the human AV node into the 3D model (Plappert et al., 2022), thereby making it suitable for evaluating fibrillatory effects on $F_{HR}$ .

## 5 Conclusion

The findings of this study suggest that elevated and reduced sympathetic activity following HUT and HDT, respectively, could contribute to the increase and decrease in $F_{\text{f}}$ measured in psAF patients. Parasympathetic activity, assessed by the magnitude of $\Delta F_{\text{f}}$ , could exert a modulating role on the effects of sympathetic activity.

## Data Availability

The data analyzed in this study is subject to the following licenses/restrictions: The data is owned by the Department of Cardiology, Clinical Sciences, Lund University, Sweden. Requests to access these datasets should be directed to pyotr.platonov@med.lu.se.

## Footnotes

1 In the original publication Abdollahpur et al. (2022), this $\Delta F_{\text{f}}$ was denoted $\Delta f_{\text{OSP}}$ to indicate that it was estimated with the Orthogonal Subspace Projection method.
