# Supplementary material for: Tilt-induced changes in f-wave characteristics during atrial fibrillation: an experimental and computational investigation
Source: Front Physiol. 2025 Jun 13;16:1498426. doi: 10.3389/fphys.2025.1498426 (PMC12202223; doi:10.3389/fphys.2025.1498426)
Supplement: Supplementary file 2 [file DataSheet1.pdf]

Supplementary Material

Mostafa Abdollahpur[a,1], Chiara Celotto[b,c], Carlos Sanchez[b,c], Felix Plapppert[a], Sten Östenson[d], Pyotr G. Platonov[e], Pablo Laguna[b,c], Esther Pueyo[b,c], Frida Sandberg[a]

[a] *Department of Biomedical Engineering, Lund University, Lund, Sweden*
[b] *BSICoS Group, I3A and IIS-Aragón, University of Zaragoza, Spain*
[c] *CIBER - Bioingeniería, Biomateriales, y Nanomedicina (CIBER-BBN), Zaragoza, Spain*
[d] *Department of Internal Medicine and Department of Clinical Physiology, Central Hospital Kristianstad, Kristianstad, Sweden*
[e] *Department of Cardiology, Clinical Sciences and Center for Integrative Electrocardiology at Lund University (CIEL), Lund, Sweden*

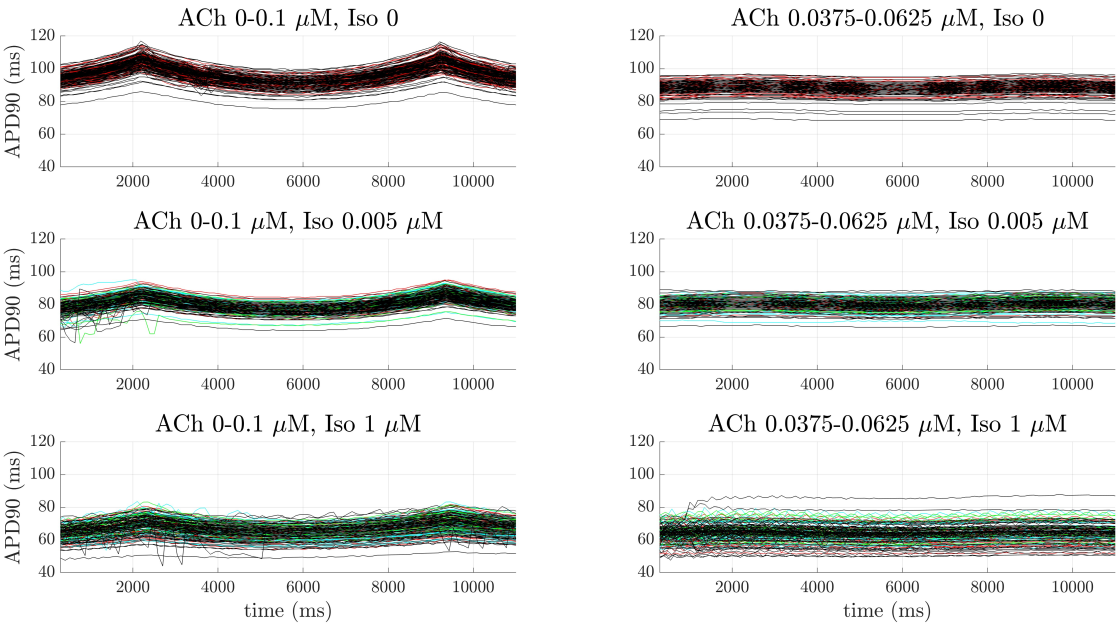

Figure 1S: APD_{90} over time measured at different tissue locations in 3D simulations. Black indicates regions without ACh or Iso; red corresponds to regions with ACh only; green to regions with Iso only; and cyan to regions with both ACh and Iso.

[1]Mostafa Abdollahpur, email: mostafa.abdollahpur@bme.lth.se

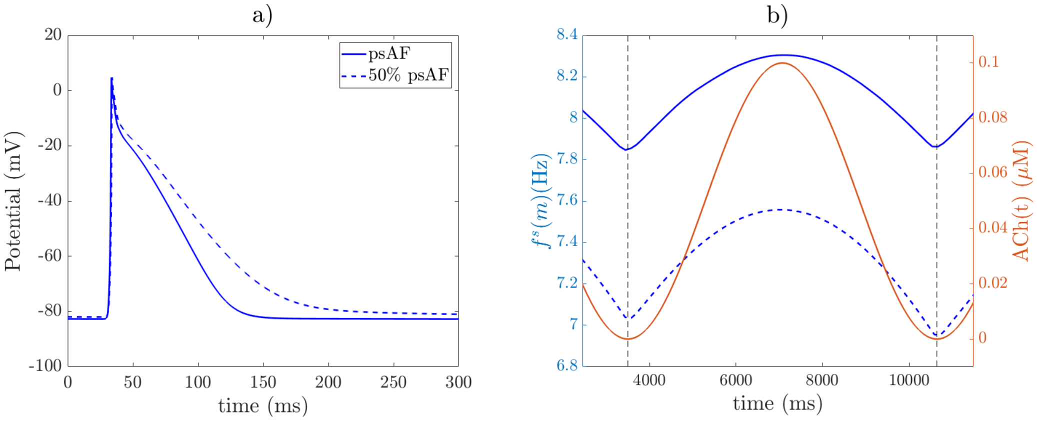

Figure 2S: Panel a) Representative action potentials at a tissue point without ACh in the psAF case (solid line) and with 50% reduced electrical remodeling (dashed line). Panel b) $f^s(m)$ (blue) and ACh(t) (red). Solid lines represent the standard psAF case, while dashed lines correspond to the case with 50% reduced electrical remodeling..
